# Review of Knowledge of Uranium-Induced Kidney Toxicity for the Development of an Adverse Outcome Pathway to Renal Impairment

**DOI:** 10.3390/ijms23084397

**Published:** 2022-04-15

**Authors:** Yann Guéguen, Marie Frerejacques

**Affiliations:** Institut de Radioprotection et de Sûreté Nucléaire (IRSN), PSE-SANTE, SESANE, LRSI, B.P. No. 17, CEDEX, 92262 Fontenay-aux-Roses, France; marie.frerejacques@irsn.fr

**Keywords:** AOP, kidney, uranium, ionizing radiation, radionuclide, heavy metal

## Abstract

An adverse outcome pathway (AOP) is a conceptual construct of causally and sequentially linked events, which occur during exposure to stressors, with an adverse outcome relevant to risk assessment. The development of an AOP is a means of identifying knowledge gaps in order to prioritize research assessing the health risks associated with exposure to physical or chemical stressors. In this paper, a review of knowledge was proposed, examining experimental and epidemiological data, in order to identify relevant key events and potential key event relationships in an AOP for renal impairment, relevant to stressors such as uranium (U). Other stressors may promote similar pathways, and this review is a necessary step to compare and combine knowledge reported for nephrotoxicants. U metal ions are filtered through the glomerular membrane of the kidneys, then concentrate in the cortical and juxtaglomerular areas, and bind to the brush border membrane of the proximal convoluted tubules. U uptake by epithelial cells occurs through endocytosis and the sodium-dependent phosphate co-transporter (NaPi-IIa). The identified key events start with the inhibition of the mitochondria electron transfer chain and the collapse of mitochondrial membrane potential, due to cytochrome b5/cytochrome c disruption. In the nucleus, U directly interacts with negatively charged DNA phosphate, thereby inducing an adduct formation, and possibly DNA strand breaks or cross-links. U also compromises DNA repair by inhibiting zing finger proteins. Thereafter, U triggers the Nrf2, NF-κB, or endoplasmic reticulum stress pathways. The resulting cellular key events include oxidative stress, DNA strand breaks and chromosomal aberrations, apoptosis, and pro-inflammatory effects. Finally, the main adverse outcome is tubular damage of the S2 and S3 segments of the kidneys, leading to tubular cell death, and then kidney failure. The attribution of renal carcinogenesis due to U is controversial, and specific experimental or epidemiological studies must be conducted. A tentative construction of an AOP for uranium-induced kidney toxicity and failure was proposed.

## 1. Introduction

The kidneys and the urinary system perform several regulatory functions, necessary for maintaining the homeostasis of the organisms, through the production and excretion of urine. The most important functions of the kidneys are the elimination of metabolic degradation products (protein metabolism), detoxification by the elimination of xenobiotics (drugs, etc.), the regulation of electrolyte concentrations (sodium, potassium, calcium, and phosphorus), the maintenance of water content and osmotic pressure, the maintenance of the acid–base and pH balances, and the biosynthesis of some hormones (such as renin, erythropoietin, and vitamin D). Their dysfunction can, therefore, lead to severe disorders that cause the individual’s general health status to deteriorate, due to acute or chronic renal failure, and eventually death. Most of the damage caused by xeno-induced renal disease is located in the proximal convoluted tubules (PCTs), an area of maximum transport, secretory, and metabolic activity. The investigation of renal function has improved greatly over the past 20 years, due to the discovery and development of new renal biomarkers [1,2,3].

Today, toxicology is moving towards a detailed characterization of the toxicity pathways to deleterious effects, using approaches such as the adverse outcome pathway (AOP) framework [4]. This concept recognizes that key events, which are causally and sequentially linked, convert the initial events that occur during exposure to stressors into adverse outcomes. AOPs have been used successfully to assess the health risks of exposure to environmental chemicals (https://aopwiki.org (accessed on 8 April 2022)) [5,6]. They make it possible to propose experimental or epidemiological scientific studies to strengthen the weak points identified in an AOP. The development of AOPs in the field of exposure to ionizing radiation or internal emitters is, therefore, a means of identifying knowledge gaps [7,8,9,10,11,12] to help prioritize research [13]. Despite the robustness of the radiation protection system, scientific knowledge regarding the risk of low doses, especially below 100 mGy, to human health is still limited. Thus, the fields of ionizing radiation and of chemical substances must deal with the difficulties in extrapolating from high to low doses [14,15,16].

Among the three main relevant adverse outcome recently reported by the Rad/Chem AOP expert group, kidney toxicity induced by uranium exposure is highlighted [17]. To put the renal biological and health effects of U exposure into perspective, compared with other renal outcomes, we developed an AOP methodology appropriate for this element, by using the current literature, and by reusing key information previously demonstrated for other exposures. Heavy metals promote similar pathways, and this review is a necessary step to compare and combine knowledge reported for other nephrotoxicants [18]. In the AOP-Wiki, several AOPs exist regarding renal failure that contain key events relevant to oxidative stress and mitochondrial dysfunction, which could be networked and added to data related to U stressors (https://aopwiki.org; accessed on 8 April 2022). Renal toxicity is the main symptom of U intoxication. It is described in experimental and epidemiological studies, as recently reviewed [19,20,21,22,23,24], but its complete AOP has not yet been proposed. This alpha-particle-emitting radioelement is naturally present on the planet, but it also has civilian (mining, milling, fuel production, reactors, and re-processing) and military uses, which increase the risk of exposure. All isotopes of U are radioactive, but due to the very long half-life of its main isotope ^238^U, both natural U and depleted U (DU) are weakly radioactive elements. Alpha particles do not penetrate beyond the outer layer of skin, and are expected to affect human health mainly after internal contamination; this impact depends partly on the route of exposure (inhalation or ingestion). Thus, U is simultaneously chemically (as a metal ion) and radiologically toxic [20,24,25,26,27]. The potential interaction of its chemotoxicity and radiotoxicity after intake must be considered in the mechanisms of action, but kidney toxicity is mainly attributed to chemical damage.

Depending on the exposure level, U induces renal tubular damage associated with kidney function impairment (e.g., decreased glomerular filtration rate and increased protein excretion) in both animals [28,29,30,31,32,33] and humans [34,35,36]. U nephrotoxicity is reported regardless of the exposure route—ingestion, inhalation, or dermal. In vitro and in vivo studies describe the mechanisms involved, and the cellular and molecular pathways responsible for these forms of toxicity, but reliable evidence has not yet demonstrated any comprehensive mechanisms of action that cause kidney impairment.

**Figure 1:**
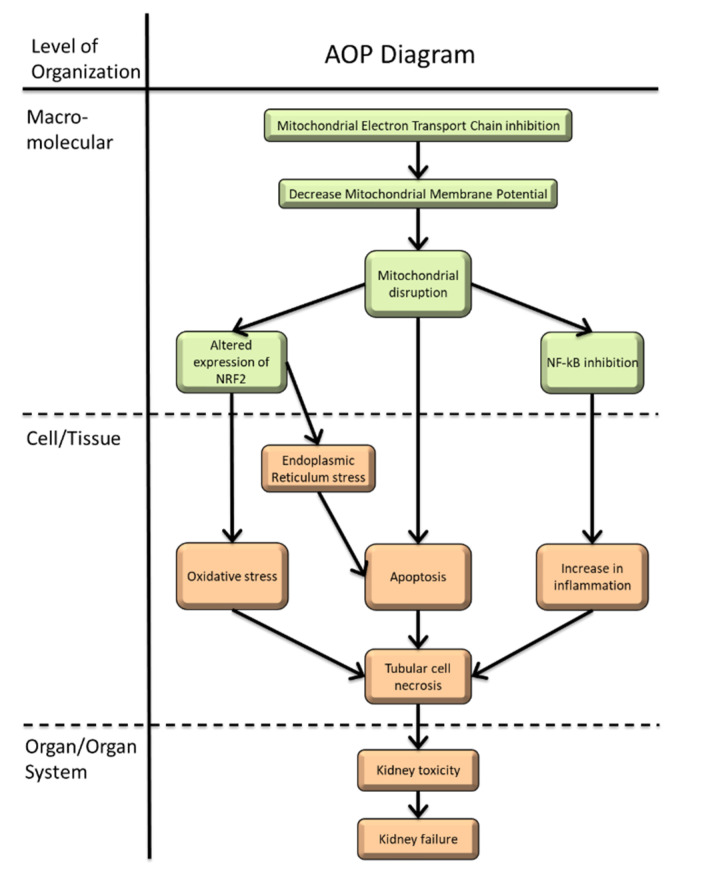
Proposed AOP diagram of kidney failure induced by inhibition of mitochondrial electron transfer chain (https://aopwiki.org/aops/447). Starting from the exposure of U and its distribution, the identified molecular pathways (molecular initiating and key events), and the cellular and tissue responses leading to adverse kidney effects.

**Figure 2:**
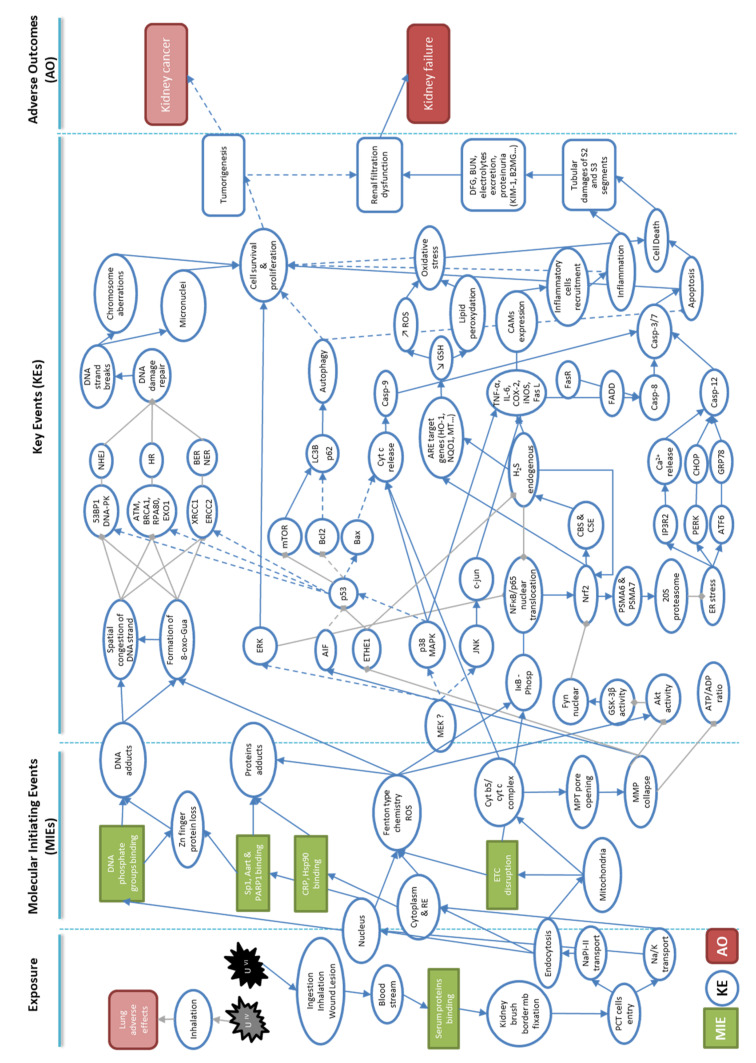
Representative network of identified molecular and cellular key events leading to adverse outcome pathways used in the development of an AOP to kidney failure in the context of U-exposure. Molecular initiating events (MIEs) are represented in green rectangles, and key events (KEs) in blue circles. Finally, the resulting adverse outcomes (AOs) are represented in red rectangles. Filled blue arrows are proven links of induction based on experimental or epidemiological studies of U nephrotoxicity, and gray segments represent links of inhibition. Dotted blue arrows represent hypothetical links that exist between the different key events.

The objective was to propose key events (KEs) and key event relationships (KERs) that formulate into an AOP, following a systematic assessment of the literature for causality, based on the Bradford Hill criteria. This enabled better classification and prediction of kidney pathogenesis, and helped to target the pathways that must be studied in future mechanistic and toxicological research, hazard identification, and risk assessments. The sections developed hereafter follow the AOP approach for kidney failure, in order to develop the first AOP proposal in the AOP-Wiki (Figure 1): from the exposure sources (anthropogenic/natural, chemical/physical forms, and isotopic composition); their routes to the organism (inhalation, ingestion, and dermal lesion); tissue/cell distribution (absorption, glomerular filtration, and cell entry); the molecular pathways identified (KEs and KERs); to the cell and tissue responses (DNA damage, inflammatory response, and apoptosis) that lead to adverse kidney effects (renal impairment and potential carcinogenesis). The relevant studies used to identify KEs or examine response–response relationships are described in the following sections and summarized in Figure 2. Non-adjacent linkages are reported by the dot line in the Figure 2.

## 2. Physicochemical Forms of Uranium

U is found in the environment in several chemical forms (including, but not limited to, oxides, chlorides, fluorides, and nitrates) and with various isotopic compositions (natural, depleted, and enriched). These are the first factors that influence the toxicity and adverse effects of U. This element belongs to the actinide family, and exhibits a range of oxidation states from +3 to +6, depending on the redox potential of the medium. U(VI) is the most soluble form, and forms complexes through the uranyl ion form UO_2_^2+^ [37,38].

The pH of water influences the solubility and absorption of U by the organism [37,38,39,40,41,42]. The acidic pH of body fluids, such as gastric juice, increases the solubilization of uranium compounds. The more water-soluble compounds (uranyl nitrate, U hexafluoride, and U tetrachloride) are more potent systemic toxicants than compounds with a poor water solubility (U tetrafluoride, sodium diuranate, and ammonium diuranate). Insoluble compounds (U trioxide or dioxide) have a much lower potential for systemic toxicity (especially renal impairment), but can affect the lungs through their long-term retention in the case of inhalation [43,44,45,46]. The cytotoxic fraction of U is a phosphate complex of uranyl (UO_2_(PO_4_)^−^ and UO_2_(HPO_4_)_aq_), as shown in the normal rat kidney proximal tubular epithelial (NRK-52^E^) cells [47,48].

U is found in different physical forms inside the cells, such as electron-dense precipitates or soluble species. Extended X-ray absorption structure spectroscopy technology has identified U precipitates such as U-phosphate, while the intracellular form of soluble U is U-carbonate [49]. U-carbonate complexes appear to be internalized in cells, and then partially metabolized into U-phosphate if U reaches the lysosomes, where low pH can induce this reaction [50].

## 3. Entry of Uranium into the Organism and the Cell

### 3.1. Absorption of Uranium into the Organism

U has three major routes of contamination: ingestion, inhalation, and through subcutaneous lesions [27,51]. In the general population, ingestion is the most common way U penetrates the organism because of its presence—natural or due to pollution—in the environment, and therefore, in food and water. Inhalation is a specific route in the occupational exposure of nuclear workers and former soldiers. Small particles of U in dust form pass through the respiratory system, reach the bloodstream [52], and then the kidneys, where they accumulate. U also reaches the bloodstream via skin lesions [52,53,54]. After ingestion, U uptake ranges from 0.1 to 2%, depending on species and solubility [46,55,56,57]. Regardless of the exposure route, U accumulates preferentially in the kidneys and the skeleton, together accounting for 40 to 80% of absorbed U through ingestion or cutaneous exposure, although inhalation into the lungs results in higher rates of total retention (up to 95%) [56,58,59,60].

### 3.2. Tissue Distribution and Reabsorption of Uranium in the Kidney

Once in the bloodstream, U, mainly found in the +6 oxidation state, combines with bicarbonate, citrate, and serum proteins. It is filtered with water as low-molecular-weight complexes through the glomerular membrane of the kidneys, then concentrates in the cortical and juxtaglomerular areas, and binds to anionic sites of the brush border membrane of the PCTs [25,61,62] (Figure 2). Albumin, IgG, apolipoprotein A1, alpha1-acid glycoprotein, alpha-2 macroglobulin, haptoglobin, transferrin, and fetuin-A are among the serum proteins with a high affinity for U (VI); fetuin-A has the highest affinity, despite its low concentration in serum [43,63,64]. Fetuin-A belongs to the cystatin family of alpha HS glycoprotein, and is involved in several functions, including bone formation, the tissue with the second highest amount of uranium accumulation.

The exact distribution of U in rat kidney tissue was determined using microbeam scanning particle-induced X-ray emission (micro-PIXE), synchrotron radiation X-ray fluorescence (SR-XRF), micro-X-ray absorption fine structure (micro-XAFS), and secondary ion mass spectrometry (SIMS). U distribution in the kidney depends on the time, dose, and chemical form of exposure. The results confirm that U is distributed mainly in the PCTs of the inner zone of the cortex, and in the outer stripe of the medulla [65,66,67,68,69]. SR-XRF and micro-XAFS show U (VI) (uranyl ions UO_2_^2+^) complexes with phosphates and carbonates in the kidney that could partially replace calcium in phosphate complexes. SIMS and micro-PIXE imaging clearly identify glomerular, distal, and proximal convoluted tubular, and the subcellular localization of U in epithelial cells. After chronic U nitrate exposure, SIMS imaging shows that U accumulates mainly in the PCTs after 12 months, but that after 18 months it is detected in all segments of the nephron, including the glomerulus [66].

### 3.3. Cellular Distribution in the Kidney

U uptake by renal proximal tubular epithelial cells occurs through diffusion or active transport. Interestingly, transmission electronic microscopy (TEM) analysis of kidney cells shows that U is taken up by endocytosis, probably through binding to plasma membrane in a nonspecific manner [49,61,70,71]. These results are confirmed by the significant correlation observed between absorptive-mediated endocytosis and U uptake in cells [72]. These authors also show a role for the sodium-dependent phosphate co-transporter (NaPi-IIa). The overexpression of NaPi-IIa in the proximal tubule cell line increases U toxicity, whereas inhibiting this co-transporter reduces it [47].

Both SIMS technology, and differential centrifugation associated with inductively coupled plasma mass spectrometry measurements, show that U enters cells in culture very rapidly (less than 15 min), probably through the diffusion of soluble forms [73,74,75]. At subtoxic concentrations (<100 µM, i.e., <50 µg/g), soluble U localizes mainly in nuclei, before it precipitates around or inside cells. U also localizes in the perinuclear regions of the cytoplasm, from 1 h after treatment to the end of a 7-day continuous exposure [73,74,76,77,78]. This nuclear and cytoplasmic distribution is also observed in rodents exposed to U via subcutaneous injection, or via the ingestion of U-contaminated water at similar tissue concentrations (1–10 µg/g) [65,79].

## 4. Molecular Initiating Events (MIEs)

The binding of U to cell macromolecules is probably the MIE that explains its cellular localization in various compartments (cytoplasm, nucleus, and mitochondria), and the first molecular targets that lead to other molecular events, such as the formation of reactive oxygen species (ROS) (Figure 1 and Figure 2).

### 4.1. Molecular Mimicry

Depending on its chemical properties, U directly binds and reacts to cytoplasmic proteins involved in the mechanisms affecting cellular integrity, such as apoptosis, inflammation, protein folding in the endoplasmic reticulum (ER), and DNA replication and repair. Uranyl ions, for example, bind heat shock protein 90-alpha, or the ERO1-like protein alpha from HK-2 cell extracts [80], whereas in silico, studies show that UO_2_^2+^ is a potent ligand of C-reactive protein (CRP), fucose binding lectin II, and mannose-binding protein C, all involved in inflammation [63,81] (Figure 2). The calcium-binding areas of the CRP protein are the main sites of UO_2_^2+^ binding; the latter prevents dead-cell recognition by CRP.

Once inside the nucleus, U causes DNA damage through two mechanisms: direct interactions with DNA, or indirectly, by generating free radicals (Figure 2). In the first case, uranyl cations interact directly with the negatively charged DNA phosphate backbone; in the second case U causes DNA damage by generating free radicals by a Fenton-type chemistry [80]. Radiotoxicity and chemical toxicity act cooperatively on DNA through both of these mechanisms. It was known for more than half a century that applying uranyl acetate solution to observe fine structure by electron microscopy produces strong nucleic acid staining [81]. More recently, studies show that uranyl ions (UO_2_^2+^) bind the nucleic acid phosphate groups of DNA and RNA, and constitute the U-phosphodiester moiety in DNA minor grooves, more specifically in the N3 position of adenine [43,82,83,84]. The direct interaction of U with DNA induces a dose and time dependent adduct formation [75,85]. The binding of DNA nucleic acid phosphate groups by uranyl ions leads to spatial congestion of the strand and trigger inhibitions of DNA repair mechanisms, as shown when uranyl acetate prevents the connection of DNA-binding proteins to DNA [86]. Moreover, the formation of uranyl-DNA adducts act as an upstream lesion that causes strand breaks, abasic sites, or possibly DNA cross-links [87], as described below (IV; molecular key events).

Zinc finger proteins play a role in DNA repair, with a zinc ion coordinated by cysteine and histidine. Cooper et al. show that U significantly disrupts zinc finger protein function, and increases DNA damage at concentrations 10-fold lower than those required to detect minimal cytotoxicity in this cell system [88], as reported for other carcinogenic and co-carcinogenic metals, such as arsenic [89,90,91,92,93,94]. Proteomic analysis of U-interacting proteins identifies the poly [ADP-ribose] polymerase 1 (PARP-1) as a candidate for U-binding in kidney cells [82], and it shows that U inhibits DNA binding of certain transcription factors, including the C2H2 zinc finger proteins Sp1 and Aart [86]. U inhibits PARP-1 activity, and non-selectively causes zinc loss from the protein. Indeed, this loss happens in all three zinc finger configurations—C2H2, C3H1, and C4ZF—involved in DNA repair pathways [88]. There is also evidence that U inhibits non-zinc finger DNA binding proteins [86].

### 4.2. Mitochondrial Damage and ROS Formation

Mitochondria are the main source of energy of the cell, providing 90% of all cellular adenosine triphosphate (ATP), and playing a significant role in apoptosis and necrosis. Mitochondrial dysfunction leads to oxidative stress and cell death, causes tissue damage, and finally kidney pathogenesis [95]. The mechanisms through which U acts on kidney mitochondria described in this section are one of the MIEs that explain the nephrotoxicity of U (Figure 1 and Figure 2). These mechanisms are also apparent in other organs, such as the liver [96,97], and the brain [98]. While this could have been developed in Section 5, on key events (KEs), for simplicity’s sake, we discuss the pathway leading to mitochondrial dysfunction entirely in this section, because it is one of the principal initiating events.

As an initial event, U impairs the electron transfer chain (ETC) at complexes II and III [99,100]. The inhibition of mitochondrial ETC leads to a decrease in MMP and ATP production. U appears to cause the collapse of mitochondrial membrane potential (MMP), as shown in mitochondria isolated from U-treated rat kidneys [99,100], and in kidney cells after in vitro U treatment [101,102]. This phenomenon is also reported in primary rat hepatocytes [96]. U sets off the concentration- and time-dependent disruption of mitochondrial outer membrane integrity and mitochondrial swelling, which is an indicator of MMP collapse [99,100]. Indeed, Pourahmad et al. report that ROS is generated by redox cycling, due to the reductive activation of U(VI) by CYP2E1, or NADPH-P450 reductase to U(V) and U(IV) [96]. Interestingly, the expression and activities of various cytochrome P450 enzymes (CYP3A1&2, 2C11, 2E1) involved in detoxifying xenobiotics [103] are impaired in the kidney or liver of U-exposed rats [104,105,106,107]; these may play a role in U cytotoxicity through oxidative stress (see Section 5 on key events).

As a consequence, U induces a decline in the ATP/ADP ratio in isolated kidney mitochondria, by decreasing the oxidative phosphorylation necessary for ATP production [99,100]. Specifically, the complexing of cytochromes (cyt) cyt b5-cyt c—known to play an essential role in the initiation of apoptosis [108]—is due to the presence of acidic residues, and the highly negatively charged heme-binding domain of cyt b5 [109,110]. Uranyl ions interfere with its formation, as Shaki et al. demonstrate: the rate of succinate (complex III substrate)-supported mitochondrial hydrogen peroxide (H_2_O_2_) production increases significantly after U treatment in isolated kidney mitochondria.

## 5. Key Events (KEs)

The effects of U on mitochondrial function, DNA conformation change, and protein folding leads to KEs that include triggering the Nrf2-pro/antioxidative, DNA damage repair (DDR), and pro/anti-apoptotic and pro/anti-inflammatory pathways (Figure 1 and Figure 2).

### 5.1. Nrf2 Pathway and Antioxidative Response

In vitro and in vivo experiments describe ROS production due to U exposure as a KE that occurs through a Fenton-type reaction, including the formation of DNA adducts, and lipid peroxidation (MDA-malondialdehyde and TBAR-thiobarbituric acid reactive substances formation) [96,99,100,111,112,113,114].

Nuclear factor erythroid 2-related factor 2 (Nrf2) is a key transcription factor in the upregulation of antioxidative enzymes responsible for synthesizing glutathione and direct-acting antioxidative molecules. The cytotoxicity of U is reported to be due to ROS formation, resulting from the inhibition of Nrf2 nuclear translocation and expression, as shown in vivo on rats exposed to a nephrotoxic dose (5 mg/kg) of U [112], or in vitro on rat NRK-52E cells treated with cytotoxic doses (400 and 800 µM) of U for 24 h [115,116]. This triggers the downregulation of the target genes, driven by the downstream antioxidative response element, including the antioxidant and the phase II detoxifying enzymes heme oxygenase 1, NAD(P)H quinone dehydrogenase 1, glutamate–cysteine ligase catalytic subunit, and thioredoxin reductase 1. It is likely that chronic exposure to U induces Nrf2 and the anti-oxidative response as an adaptive reaction to ROS formation, as shown in our previous works [65,117], whereas damage from acute high doses disrupts Nrf2 expression and nuclear translocation.

As a consequence, the levels of glutathione, a well-known antioxidant defense factor against ROS, dose-dependently decrease after uranyl acetate treatment in both isolated rat kidney mitochondria [99,100] and in kidney cells [101,112,116,118]. Moreover, the expression of the catalase (CAT) protein, an antioxidative enzyme, decreases in vitro 24 h after U treatment in HK-2 and NRK-52^E^ cells [101,116], and also in vivo in rat kidneys [111,112,118]. In mouse kidneys, this causes a lower expression of SOD, an enzyme known to trigger CAT activity and an antioxidative response expression. In turn, this exacerbates the decrease in CAT activity, and thus, increases lipid peroxidation [32,116,119].

Interestingly, U injected into rats decreases the expression levels of cystathionine β-synthase (CBS) and cystathionine ɣ-lyase (CSE), two enzymes that catalyze the endogenous formation of hydrogen sulfide (H_2_S) [112,116]. H_2_S is an endogenous signaling gaseous signaling molecule that regulates antioxidative, anti-inflammatory, and cytoprotective responses [120]. The decreased H_2_S renal concentration triggers a modification of the Nrf2 pathway, which, in turn, leads to decreased antioxidative enzyme activity [112]; H_2_S induction by a drug treatment in kidney cells, and in U-exposed rats, re-establishes the Nrf2 active pathway, and suppresses the oxidative stress resulting from the U treatment [112,115,116]. The U-induced inhibition of Nrf2 expression and nuclear translocation initiates the down-expression of CBS and CSE, reducing endogenous H_2_S formation and reinforcing the inhibition of Nrf2 [116]. In vitro studies show that CBS and CSE expression is regulated by Nrf2 [121,122]. Zheng et al. hypothesize that the U-induced decrease in Nrf2 expression is due to the inhibition of the upstream regulator protein kinase B (Akt), and glycogen synthase kinase 3 beta activities, which increase the nuclear expression of the tyrosine-protein kinase Fyn [112]. This nuclear overexpression down-regulates Nrf2, and therefore, triggers the inhibition of antioxidant enzyme transcription, and leads to oxidative stress in kidney cells.

### 5.2. Endoplasmic Reticulum (ER) Stress Response and Ca^2+^ Homeostasis

The ER is a major reservoir of intracellular calcium, similar to the mitochondria, and its efflux into the cytosol is a common response to many toxic agents. Some studies indicate that U toxicity is partly explained by a perturbation in calcium homeostasis.

U-induced ER stress is partly due to the inhibition of Nrf2 nuclear expression [115]. These authors show that in NRK-52^E^ cells, Nrf2 promotes the expression of the proteasome subunit alpha type-6 and beta type-7, both of which contribute to the assembly of the 20S proteasome complex. But, as shown in the preceding subsection, U diminishes Nrf2 nuclear translocation, and therefore, reduces the formation of this complex, creating ER stress. On the other hand, the higher ROS level, due to U intoxication, significantly increases the expression of glucose-regulated protein 78, and C/EBP homologous protein (known as CHOP) [115]. These transcription factors are induced respectively by the activating transcription factor 6, and protein kinase RNA-like ER kinase (known as PERK). The activation of the unfolded protein response due to ER stress then activates caspase-12, and initiates the apoptosis cascade.

In a U-exposed mouse kidney, the expression of the IP3 receptor 2 (IP3R2) and sarco/ER Ca^2+^-ATPase pump (known as SERCA) increase, but type 1 IP3Rs are not affected [123]. By contrast, IP3-induced calcium release rapidly decreases in isolated membrane vesicles of the mouse kidney, and probably induced positive feedback on IP3R2 and SERCA2 expression. IP3R2 also plays a role in U-induced apoptosis (from 50 μM), as reported by the same group in the HEK293 cell line [124]. Exposure to U could, therefore, increase cellular apoptosis through IP3R2 expression. This modification of calcium homeostasis is underlined by the changes in numerous gene expression (notably by an elevation of IP3 kinase mRNA) during a transcriptomic study of U-exposed HEK293 cells (Prat et al., 2005).

### 5.3. DNA Damage Response

U causes DNA damage by interacting directly with DNA or DNA proteins, or indirectly through the release of ROS, and the formation of DNA adducts.

Two DNA double-strand break (DSB) repair mechanisms—homologous recombination (HR) and non-homologous end-joining (NHEJ)—are impaired by U exposure, shown by the low concentration of 10 µM, 24 h after treatment of the bronchial epithelial cell line (BEP2D) [125]. This exposure inhibits critical DDR proteins such as ATM, BRCA1, RPA80, and EXO1 (all using the HR mechanism), as well as DNA-PKcs and 53BP1 proteins (by the NHEJ pathway); it ultimately leads to the dose-dependent accumulation of nuclear DSBs, as shown by ɣ-H2AX [125]. In a zebrafish model, U also impairs DNA-PK kinase activity [126].

Interestingly, Yellowhair et al. show that deficient base/nucleotide excision repair (BER and NER) mechanisms are directly related to U-induced cytotoxicity in vitro [75]. A BER-deficient EM9 CHO cell line experienced greater U cytotoxicity than the H9T3 cell line cloned with the XRCC1 gene, which is involved in the BER mechanism. Similarly, U cytotoxicity is higher in the UV5 CHO cell line deficient in NER than the 5T4-12 cell line cloned with the ERCC2 gene, involved in the NER mechanism. These results imply that DNA, BER, and NER pathways participate in the response to U exposure, and are directly correlated with U-induced DNA damage [75]. DNA damage and DDR deficiency explain, at least in part, the U-induced genotoxicity and cytotoxicity described below.

### 5.4. Pro/Anti-Apoptotic Pathways

The induction of signaling pathways involved in U-related cell survival, or apoptosis, is mainly due to redox imbalance, and the aforementioned mitochondrial modification. The fact that in NRK-52^E^ cells apoptosis peaks when MMP is at its minimum (decreased by a factor of 60 compared to controls) shows that MMP reduction is an event upstream of mitochondrial apoptosis [102] (see Section 3 for the mechanisms of mitochondrial disruption). This mitochondrial dysfunction partly explains the modulation of PI-3K, MAPK, Bax[Bcl-2 associated X protein]/Bcl2, and Fas/Fas L pathways, through direct or indirect mechanisms, as reported below and shown in Figure 2.

#### 5.4.1. From PI-3K Pathway to Cell Survival

In NRK-52^E^ cells, prosurvival Akt pathway signaling decreases in the short term, 18 h after uranyl acetate exposure [115], but is induced in the long term, as shown after a single injection of 2 or 4 mg/kg uranyl nitrate in Swiss albino mice [127]. Three days after exposure, the prosurvival signaling molecule extracellular signal-regulated kinase increases in their kidneys, with a transient elevation of phosphorylated Akt levels seven days after injection.

#### 5.4.2. From MAPK Pathway to Apoptosis

DU-induced apoptosis is correlated to the augmentation of p38-MAPK phosphorylation and activation [128], modifying cyt-c release, and caspase-9-induced apoptosis [129]. U increases cyt-c translocation from the mitochondrial to the cytosolic fraction in both the rat kidney [99,100] and human kidney cells [101]. U also triggers the cytoplasmic concentration of the apoptosis inhibitor factor (AIF) in human kidney cells [101], which can lead to both caspase-dependent and caspase-independent apoptotic pathways [130]. U-induced apoptosis is also activated by the inhibition of the anti-apoptotic protein ethylmalonic encephalopathy 1 (ETHE1) enzyme, a mitochondrial sulfur dioxygenase, which most likely translocates to the cytoplasm due to mitochondrial swelling, to inhibit p53-induced apoptosis [131] through the regulation of mTOR pathway [132]. The authors report that an increase in nuclear p53, associated with a decrease in intracellular phosphorylated mTOR, is observed with an increasing DU dose (125–500 µM) in the HEK293 cell line.

Moreover, Sangeetha et al. show that the activation of the pro-apoptotic c-Jun N-terminal kinase (JNK) is also induced, and peaks 72 h after exposure in mice. These results suggest this activation is partly responsible for U-induced apoptosis. Further confirmation is evident in similar results for other cell types, such as alveolar macrophages [128] and hepatocytes [114].

#### 5.4.3. Bax/Bcl2 Pathway

The mitochondrial disruption induced by U is also related to the regulation of some anti-/pro-apoptotic factors, specifically B-cell lymphoma 2 (Bcl-2), and Bcl-2-associated X proteins. U induces a decrease in the mitochondrial concentration of the Bcl-2 protein in HK-2 cells treated with 500 µM of DU for 24 h, as well as the overexpression of the Bax level [101]. These KEs of apoptosis are also identified in vivo in the kidneys of mice treated with a single dose of 10 mg/kg U, 4 days after an IP injection [118].

#### 5.4.4. FasR/FasL Pathway

The FasR (Fas receptor) pathway is also induced by U in vitro, shown by the upregulation of sFasL (Fas ligand) by 400% after treating HK-2 cells with 500 µM of U. This result suggests that the FasR/caspase-8 pathway is involved in the U apoptotic effect in the kidneys [101]. Thus, both mitochondrial and FasR-mediated pathways are involved in U-induced apoptosis in HK-2 cells.

### 5.5. The NF-κB Pathway

The nuclear transcription factor kappa-light-chain-enhancer of activated B cells (NF-κB) is a critical mediator of cellular responses to inflammation, oxidative stress, and cell survival. NF-κB and phosphorylated IκB are activated in the kidneys of rats injected with single doses of 2.5, 5, or 10 mg/kg of uranyl acetate [112]. U decreases the formation of H_2_S [112,116], an endogenous gas exerting antioxidative, anti-inflammatory, and cytoprotective effects in the kidneys. NaHS treatment, along with U contamination, prevents NF-κB/p65 nuclear translocation in renal cells, and thus, inhibits the protein expression of NF-κB-activated inflammatory genes, including tumor necrosis factor-alpha (TNF-α), nitric oxide synthase (iNOS), and cyclooxygenase 2 (COX-2). These results indicate that U activates the NF-κB pathway, and thereby induces inflammation, probably by reducing endogenous H_2_S. U alters ETHE1, a mitochondrial enzyme catabolizing H_2_S [131], an event that may initiate this pathway.

### 5.6. Metallothionein

Metallothioneins (MTs) are low-molecular-weight proteins, with a metal-binding function that involves them in the detoxification of heavy metals and essential metal homeostasis. By interacting with zinc ions, they protect cells from oxidative stress, or apoptosis caused by U exposure to HK-2 or HEK293 kidney cells [101]. MT^−/−^ mice are more susceptible to U-induced nephrotoxicity, which decreases their levels of antioxidant enzymes, sodium–glucose cotransporter (SGLT), and sodium–phosphate cotransporter (NaPi-II); it increases ROS, lipid peroxidation, and pathological kidney damage [118]. The same group shows that dysregulation is likely a key downstream event for the induction of the apoptotic pathways [131].

### 5.7. DNA Damage and Genotoxicity

U exposure causes DNA damage and mutations in cells [85,133,134], most likely by impairing the DDR pathways, as described in Section 5.3. In this section, the focus is on DNA strand breaks and genotoxicity as the consequences of DDR alterations.

Most studies of U genotoxicity show that the DNA damage induced in kidney cells by U exposure can be attributed to U chemical toxicity, rather than its radioactivity [19]. Some studies, however, investigate the role of radiation by increasing the specific activity of U (enrichment of isotope ^235^U), but at equimolecular U concentration in non-kidney cells. Miller et al. show that in an osteoblast cell line, U is more mutagenic when composed of higher amounts of ^235^U isotope (higher specific activity); this sets off an increase in the frequency of hprt mutants [26,135]. In fibroblast cells, the genotoxicity of U increases with the radiation dose, as observed in a study showing a stronger clastogenic effect with enriched U compared to DU [136]. Nevertheless, the aneugenic effect of DU also increases significantly with U exposure.

#### 5.7.1. DNA Strand Breaks

U induces both single-strand breaks and DSB in vitro. As explained above, in Section 4 (MIEs), the genotoxicity of U results from either free radical production, or the direct binding of U to the negatively charged phosphate backbone of DNA, which leads to DNA adduct formation [19,75,80,137,138,139]. The DNA damage induced by U is activated by the presence of ascorbate (vitamin C), probably due to the direct reaction of the uranyl ascorbate complex with the negatively charged DNA phosphate backbone, which subsequently promotes uranyl-DNA adducts [80,87,140].

As guanosine is the ribonucleoside with the lowest redox potential [141], guanine is an electron donor for uranyl ions. The resulting guanine cation passes through the DNA strand, moving from DNA guanines to others DNA bases. This process triggers guanine residue oxidation, which is one mechanism for the formation of 8-hydroxydeoxyguanosine (8-OHdG), a commonly used marker of oxidative stress-derived DNA damage by U [142]. In addition, 8-OHdG is directly generated by ROS, through the exchange of ions between hydrogen peroxide and uranyl ions [137,143]; it can cause oxidative damage to DNA in HEp-2 cells [144]. U also induces 8-OHdG dose- and time-dependently in vitro, and is partially inhibited by the antioxidant enzyme superoxide dismutase (SOD) [138].

Studies of ɣ-H_2_AX foci and the comet assay show that U dose-dependently induces DSBs within 24 h after the treatment of kidney cells, leading to cell death [102]; the same is true in the BED2D bronchial cell line [125]. Rat exposure to U through inhalation induces DNA strand breaks in both kidney and bronchoalveolar lavage (BAL) cells [145,146]. The comet assay reveals that some of the DNA damage in these cells is made up of DSBs, and thus, reinforces the hypothesis that U-alpha radiation contributes to its genotoxic effects in vivo [146], and in vitro [26,135].

#### 5.7.2. Chromosomal Aberrations and Micronuclei Formation

U genotoxicity is described in lung epithelial cells or macrophages, and is reported to be caused by two different mechanisms: one clastogenic by chromosome disruption or breakage; and the other aneugenic, by inducing aneuploidy (an abnormal number of chromosomes) [136,147,148]. These in vitro studies show that U acts as a cell proliferation suppressor, induces elevated micronuclei, chromosomal aberrations, and lesions in both CHO cells and in human bronchial epithelial cells [148,149,150,151]. Other studies show that U causes genomic instability and gene mutations [26] that increase the death rate in immortalized human osteoblasts [151]. The formation of micronuclei is not, however, observed in NRK-52^E^ cells, even after a U treatment of as much as 600 µM [102].

Higher numbers of chromosome aberrations and lesions are observed in populations living or working in U-contaminated areas, but the association with U exposure is difficult to demonstrate [152]. Epidemiological studies with biological analyses of DNA damage or genomic instability in U workers have recently been reviewed elsewhere [24].

### 5.8. Apoptotic and Necrotic Endpoints

U-induced cell deaths are mainly described as either apoptotic or necrotic in cell cultures, and in tissue observations of exposed animals. Oxidative stress and DNA damage induced by U exposure triggers cytotoxicity when the cellular mechanisms of protection are overwhelmed.

U-induced apoptosis is observed in both in vitro and in vivo studies, in renal tissue and renal cells, but also in other tissues [73,76,96,99,153,154,155]. This apoptotic activity is often attributed to lysosomal or mitochondrial alterations, and increased ROS production [96,99,102]. Interestingly, U induces the activation of both caspase-3 and caspase-9 in NRK-52^E^ cells in a concentration- and time-dependent manner [102,115], whereas it triggers the activation of caspase-8, as well as caspase-3 and caspase-9, in human kidney HK-2 cells [101]. Caspase-3 activation by caspase-9 involves a mitochondrial (intrinsic) apoptotic pathway, whereas the activation of caspase-3 by caspase-8 triggers a non-mitochondrial (extrinsic) pathway, as described in Section 4. In vitro, there is probably a threshold of around 400–500 µM beyond which damage becomes irreversible, and leads to cell death. ROS and ER stress trigger the induction of pro-apoptotic proteins. Indeed, Yi et al. show that U induces ER stress in kidney cells, leading to the activation of caspase-12 and caspase-3 within 18 h [115]. In vivo, U-induced apoptosis (TUNEL-positive nuclei) are confirmed in male Swiss albino mice kidney cells, after IP injections of 2 and 4 mg/kg of U, starting from 72 h after treatment, and continuing until 14 days post-treatment [127]. A single IP injection starting at 2 mg/kg U induces the expression and activity of the apoptosis-effecting caspases 3/7 in rodent kidneys [118,156].

Necrosis is attributed to the increased release of gamma glutamyl transferase (GGT), lactate dehydrogenase (LDH), and acid phosphatase enzymes, all reported in the urine of U-exposed animals [32,119]. In vitro, U exposure results in increased LDH and N-acetylglutamate concentrations, both markers of decreased viability, in various kidney cell lines: HK-2 [101], HEK293 [76,155], LLC-PK1 [42], and NRK-52^E^ [40,102,116]. Thus, this kind of cell death is influenced by the concentration and duration of U exposure, the chemical speciation of the U, and pH [40,157].

### 5.9. Inflammation

Inflammation is described as a mechanism of toxicity after the injection, inhalation, or ingestion of U [146,158,159,160], but publications about its involvement in U nephrotoxicity are sparse [112,115,159], despite the major role that inflammatory processes play in kidney injury [161].

In acutely U-exposed rats, an inflammatory response is reported to be due to NF-κB activation, and the upregulation of three inflammatory genes (TNF-α, iNOS, and COX-2) in rat renal homogenates [112]. The overexpression of genes involved in inflammation, such as osteopontin (Opn), Pecam, or Gal-3, is also observed in mouse kidneys exposed to U in a transcriptomic study [159]. Moreover, a single injection of U in mice induces the inflammatory upregulation of intercellular and vascular cell adhesion molecules (ICAM and VCAM), which are involved in the recruitment of inflammatory cells to the kidneys [156]. However, it is not clear from these results whether the inflammatory genes are induced by epithelial cells, inflammatory cells, or vascular cells because they are obtained on kidney homogenates. Nevertheless, Opn is a protein involved in inflammation and mineralization regulation; its expression is modified after exposure to U in the HK-2 cell line [162,163]. The authors hypothesize that a decrease in Opn in epithelial cells is related to the altered p38 MAPK pathway, and contributes to a delayed inflammatory reaction, several days after U exposure.

In comparison, the lungs of mice exposed to U by inhalation reveal a rapid and brief increase in the production of the pro-inflammatory cytokines TNF-α, IL-8, and MIP-2 [146]. In vitro, Gazin et al. demonstrate that the secretion of TNF-α, a pro-inflammatory cytokine, is due to the U-induced activation of JNK and p38-MAPK signaling pathways in macrophages [128]. Interestingly, both of these signaling pathways are also partly responsible for U-induced apoptosis, as described above. This suggests that a parallel activation by TNF-α occurs between these mechanisms, leading to cell death (Figure 2).

### 5.10. Autophagy

Autophagy is a component of the adaptive response of the cell; induced by starvation or stressful conditions, it promotes cell survival by recycling damaged proteins and organelles. Very few studies investigate its role in U nephrotoxicity. U can trigger autophagy rapidly in osteoblastic and osteocytic cells, by upregulating the level of the membrane-bound, microtubule-associated protein 1 light chain 3 protein (LC3-II), a marker of autophagosome formation [164,165]. Nonetheless, 24 h after the U treatment of the UMR-106 osteoblastic cell line, inhibition is observed of the autophagic flux, capable of disturbing the mineralization capacity of osteoblasts and osteocytes.

To date, few data on autophagy in kidney cells or tissue after U exposure are reported [132,166]. Following a single IP injection of 5 mg/kg, U in mouse kidneys is able to induce, 72h later, the protein expression of LC3-II in situ, a marker of autophagosome formation, whereas exposure to fluoride, another nephrotoxicant, did not induce LC3-II expression at a similar nephrotoxic dose [166]. In vitro, others show that LC3-II rapidly increases at U toxic level, probably due to the inactivation of mTOR pathway [132].

## 6. Kidney Adverse Outcomes (AO)

It is well-known that U induces tissue lesions and dysfunction in the kidney, the organ most sensitive to U intoxication, and that this can lead to acute or chronic kidney disease [25]. Chronic oxidative stress, inflammation, DNA damage, and cell death are important contributors to the U-induced renal impairment of the S2 and S3 segments (Figure 2) [24,30,58,167,168,169].

In experimental studies, U exposure triggers modifications in such renal function indicators as serum creatinine and blood urea nitrogen (BUN) [118,170,171,172]. These changes indicate renal impairment and the dysfunction of the filtration function of the kidneys, shown by reduced creatinine clearance, which is an indicator of the glomerular filtration rate [30,32]. Impairment of renal function in rodents is confirmed by elevated urinary levels of glucose, proteins, and electrolyte excretion (sodium, potassium, magnesium, calcium, and inorganic phosphate), probably due to decreased reabsorption, or the perturbation of transport properties of renal cells from the proximal tubules [30,32,156,159,173].

Moreover, U induces a significant increase in urine volume, alkaline phosphatase, β_2_-microglobulin, LDH, and N-acetylglutamate in exposed rodents; these changes indicate tubular damages and probably a defect in water reabsorption [58,112,173,174]. Indeed, the transmembrane protein KIM-1, a sensitive biomarker of PCT injury, is induced in the kidney of mice or rats treated with a single IP injection of DU at 2–5 mg/kg [156,175]. Tubular and glomerular damage, observed by histological analyses, depends on the dose administered, beginning one hour after exposure in rodents at a high level [20,25,30,33,58,176]. Renal tubular epithelial cell hyalinosis, cell vacuolization, cell shedding, necrosis, and urinary casts are observed in rodent kidneys after acute U exposure [58,177].

The inflammatory response, and its role in renal impairment, are investigated far less often, even though one previous study suggests the involvement of an infiltrate composed of mononuclear cells [30]. As described in the previous section, the upregulation of pro-inflammatory cytokines in the kidneys is probably induced by activating MAPK and NF-κB pathways. It was recently shown that the overexpression of adhesion molecules (CAMS) after U exposure [156,178] would result in the recruitment of inflammatory monocytes or macrophages. Renal impairment associated with U accumulation in the kidney is not always detected after chronic U exposure [28,29,30], although this may depend on the duration of exposure and the dose [65,179,180]. Finally, U directly interacts with proteins involved in the immune response, such as CRP, or indirectly initiates and promotes inflammation in the kidney, which leads to the progression step.

Several experimental studies describe U as carcinogenic, depending on the route of exposure and the solubility of the compound, but the debate about whether it meets the criteria for a kidney carcinogen remains open [19,181]. Carcinogenesis is described as a three-step event [181]. The initiating event is the modification of the DNA of kidney cells through direct and indirect mechanisms [19,75,80,137,138,139], transforming them from normal to potentially tumoral cells. As DDR mechanisms are then impaired after U exposure, nuclear DNA alterations probably accumulate, making it possible for them to proliferate [148,149,150]. As shown in the KEs section, the p53 pathway alteration is also involved in tumorigenesis to favor cell survival and proliferation.

## 7. Population Outcomes: Kidney Dysfunction and Tumorigenesis

Renal biomarkers are measured in some of the epidemiological studies of nuclear workers exposed to various types of U, military personnel exposed to DU, and the general population exposed to natural U. There are some informative reported cases of acute human exposure to U after deliberate ingestion, accidental burn or dermal exposure, inhalation, treatment by intravenous injection of various chemical forms (uranyl nitrate, acetate, U oxide or tetrafluoride, or hexafluoride), reviewed in several reports [182,183]. The renal effects observed range from renal tubular dysfunction to acute renal failure, manifested by, among others, glucosuria, proteinuria, and albuminuria, with a gradual return to normal after several weeks or months. These changes in renal biomarkers among people overexposed to U may be due to tubular necrosis, which is the most relevant clinical consequence of acute U toxicity in humans [184]. Although such acute exposure clearly results in kidney injury, long-term exposure studies are less conclusive when demonstrating the induction of kidney diseases in exposed general, occupational, or military populations, as reviewed previously [27,185,186,187,188,189,190], due to methodological limitations such as low statistical power, suboptimal control for potential confounders, and unsatisfactory characterization of U exposure [191,192].

Nevertheless, epidemiological studies of populations with chronic or repeated U exposure report changes in urinary or serum clinical biomarkers (glucosuria, calcium, and phosphate urinary excretion), indicating potential tubular damage in populations consuming well water with elevated U concentrations (up to 781 μg/L) [24]. It is difficult to compare human and rodent studies because the route of exposure, level of intake, and duration of exposure differs. Nevertheless, with an assumed uncertainty factor of 100 (for inter- and intraspecies variations) used to calculate the WHO guideline value for U (30 µg/L) for a 60-kg adult drinking 2 L of water per day, the body burden of U in rodents is at least an order of magnitude lower than the range of nephrotoxicity (2–5 mg/kg/d), if administered acutely [20,180,193]. The absence of any modification of creatinine clearance or proteinuria in the general population exposed to natural U indicates the lack of potential glomerular or tubular damages [36,194]. Nonetheless, Zamora et al. (1998) observe the induction of alkaline phosphatase (ALP) and β2-microglobulin (B2MG) excretion in urines—both biomarkers because of tubular damages and membrane disruption [36]. B2MG secretion is also induced in individuals drinking water contaminated with 0.2 to 470 µg/L; their urinary U concentrations are up to 8 times higher than normal, although no nephrotoxicity is observed [195].

Studies of occupational exposure, and of Gulf War veterans, also report high levels of urinary or serum B2MG, or retinol-binding proteins, as markers of PCT function, for workers or military personnel with U concentrations in urine of 65 to 1978 µg/L [22,35]. Nonetheless, studies do not show an increased risk of death from nephritis or other renal diseases, due to the wide confidence intervals in these cohorts (reviewed by [22,196,197,198,199,200]), except very recently for kidney cancer [201,202]. This single epidemiological study shows a significant radiation dose–response relation for kidney cancer (RR 1.92 at 100 mGy) in U-exposed processing workers [202]. On the other hand, a monotonically increasing, but non-statistically significant, risk of kidney cancer mortality is observed in U enrichment workers [201]. Thus, the association between U exposure and kidney cancer remains controversial in epidemiological studies [203,204].

Experimentally, the few studies of kidney cancer occurring during U exposure observed slight fibrosis in rats [28,167], but no induction of renal tumors [205], except in one study of mice exposed to highly radioactive ^233^U; they also developed bone and liver cancer, and myeloid leukemia [168]. Due to the very low incidence rate of renal tumors in humans and animals, the small number of animals used does not provide sufficient statistical power.

## 8. Conclusions

This review, based on an AOP approach, proposed a blueprint of uranium-induced toxicity in the kidney, and allowed us to identify the different steps and gaps between the molecular initiating events to the impairment and the dysfunction of the kidney that could lead to chronic kidney diseases and kidney cancer in experimental models or in humans. Most of the studies described above related to MIEs and KEs, and the response–response relationships identified using uranium studies to specific endpoints.

The large amount of mechanistic knowledge generated by in vivo and in vitro studies must be incorporated into the health risk assessment of ionizing radiation. Such an implementation has been proposed in the past, in the form of mathematical models of radiation-induced carcinogenesis based on biological mechanisms [206]. This approach, however, has found only an extremely limited application in radiation protection up to now [8,9,10]. The AOP approach, on the other hand, is widely used to assess the health risks of exposure to chemicals. Moreover, several epidemiological studies show an association between chronic exposure to low doses of ionizing radiation and the onset of cancers [207,208]. However, there is no scientific consensus about the existence and nature of the mechanisms associated with the cancer process linked to chronic or repeated exposure to ionizing radiation, or internal emitters at low doses (<100 mSv).

The concept of AOP, therefore, shows promise for enabling the consolidation and integration of a large amount of knowledge obtained from both epidemiology and biology, with a view towards improving the calculation of the radiological health risks. It is also expected to strengthen the extrapolation between the effects of different exposures, and perhaps even to support the prediction of the effects of mixtures [209]. Ultimately, we hope that this review will pave the way for AOP networks to develop for other exposures to ionizing radiation, and thus, contribute to the improvement of the radiation protection system.

## Abbreviations

8-OHdG	8-hydroxydeoxyguanosine
Akt	regulator protein kinase B
ALP	alkaline phosphatase
AOP	adverse outcome pathway
Bax	Bcl-2 associated X protein
Bcl-2	B-cell lymphoma 2
CBS	cystathionine β-synthase
BER	base excision repair
B2MG	β2-microglobulin
CAT	catalase
CKE	cellular key event
CRP	C-reactive protein
CSE	cystathionine ɣ-lyase
COX-2	cyclooxygenase 2
cyt c	cytochrome c
DDR	DNA damage repair
DSB	double-strand break
DU	depleted uranium
ER	endoplasmic reticulum
ETC	electron transfer chain
H2S	hydrogen sulfide
HR	homologous recombination
iNOS	nitric oxide synthase
IP	intraperitoneal
IP3R2	IP3 Receptor 2
JNK	c-Jun N-terminal kinase
micro-PIXE	microbeam scanning particle-induced X-ray emission
micro-XAFS	micro-X-ray absorption fine-structure
MIE	molecular initiating event
KE	key event
MMP	mitochondrial membrane potential
MT	metallothionein
NaPi-IIa	sodium-dependent phosphate co-transporter
NER	nucleotide excision repair
Nox4	NADPH oxidase 4
NF-κB	nuclear transcription factor kappa-light-chain-enhancer of activated B cells
NHEJ	non-homologous end-joining
Nrf2	nuclear factor erythroid 2-related factor 2
NRK-52E	normal rat kidney proximal tubular epithelial (cell line)
p38 MAPK	p38 mitogen-activated protein kinase
PARP-1	poly [ADP-ribose] polymerase 1
PCT	proximal convoluted tubule
ROS	reactive oxygen species
SIMS	secondary ion mass spectrometry
SOD	enzyme superoxide dismutase
TEM	transmission electronic microscopy
U	uranium
